# Critical areas for sea turtles in Northeast Brazil: a participatory approach for a data-poor context

**DOI:** 10.7717/peerj.17109

**Published:** 2024-03-25

**Authors:** Yedda Christina Bezerra Barbosa de Oliveira, Douglas Nazareth Rivera, Luciano Carramaschi de Alagão Querido, José da Silva Mourão

**Affiliations:** 1 Programa de Pós-Graduação em Etnobiologia e Conservação da Natureza, Universidade Federal Rural de Pernambuco, Recife, Pernambuco, Brazil; 2 Programa de Pós-Graduação em Conservação da Fauna, Universidade Federal de São Carlos, São Carlos, São Paulo, Brazil; 3 Centro de Estudos Integrados da Biodiversidade Amazônica, Instituto Nacional de Pesquisas da Amazônia, Manaus, Amazonas, Brazil; 4 Departamento de Biologia, Universidade Estadual da Paraíba, Campina Grande, Paraiba, Brazil

**Keywords:** Participatory GIS, Local ecological knowledge, Ethnobiology, Small-scale fishery, Conservation

## Abstract

Fishing is one of the main threats to sea turtles due to the risk of entanglement in lost nets, vessel collision and mortality due to incidental catches. In Brazil, most of the studies regarding fishing interactions with sea turtles are focused on pelagic longline fisheries in the South and Southeast regions. However, their main reproductive areas in Southwest Atlantic RMU occur in Northeast Brazil, which overlaps small-scale coastal gillnet fisheries. Here, we aimed to use ethnobiology and participatory approaches as simple and cost-effective methods to identify areas for sea turtle conservation where impacts from small-scale fisheries are most likely. Expert captains were trained to recording sea turtle sightings during navigations from the landing port to the fishing grounds, informing their folk nomenclatures. By interpolation of environmental data (chlorophyll and bathymetry) and ecological data, we predicted habitats of 3,459.96 km² for *Caretta caretta*, *Chelonia mydas*, and *Eretmochelys imbricata* and fishing zones of 1,087 km² for management in 20 m and 50 m depths. Our results contributes to ongoing discussions of bycatch mitigation for sea turtle species and identification of habitats. We highlights the importance of considering particularities of overlapped areas in marine spatial planning and co-management arrangements.

## Introduction

Fishing is one of the main threats to ecosystems, exerting pressure not only on target species for coastal and oceanic fisheries (*i.e*., overfishing), but also on species with no commercial value (*i.e*., bycatch) (ICMBIO, 2016; Sandoval Gallardo et al., 2021; Karnad, 2022). The impacts of fishery activity on sea turtles, as with most marine megafauna, are often the accidental capture in gillnets, lines and hooks, and collision with vessels, which results in mortality (Lewison et al., 2004; Fuentes et al., 2023). Large regions of the ocean where industrial fisheries overlaps sea turtle distribution have been well understood but it is not the same for small-scale fisheries (Kroodsma et al., 2018; Sequeira et al., 2019). The lack of technology for identifying, tracking and monitoring artisanal boats is the main limitation (Sequeira et al., 2019).

One conservation strategy for species with a wide distribution, such as sea turtles, is to define segments of populations spatially that are exposed to similar anthropogenic threats and biogeography (*i.e.*, regional management units or RMUs; Wallace et al., 2010, 2023). Five species of sea turtles inhabit the Southwest Atlantic RMU in migratory corridors, feeding grounds and nesting areas: green turtle (*Chelonia mydas*), hawksbill (*Eretmochelys imbricata*), loggerhead (*Caretta caretta*), leatherback (*Dermochelys coriacea*), and olive ridley (*Lepidochelys olivacea*). Conservation status ranges from “least concern” for *C. mydas* to “critically endangered” for *E. imbricata* locally (ICMBIO, 2016), and from “vulnerable” for *C. caretta*, *D. coriacea* and *L. olivacea* to “endangered” for *C. mydas* globally (IUCN, 2023). This RMU comprises Brazil, Argentina and Uruguay, where species occur in more than one life stage under high mortality rates (Cantor et al., 2020).

Most of the studies regarding fishing interactions with sea turtles in Brazil are about pelagic longline and industrial trawling fisheries, in the subtropical areas of the South and Southeast region (Fiedler et al., 2012; Schroeder et al., 2016; Duarte, Broadhurst & Dumont, 2019; Lopez-Mendilaharsu et al., 2019; López-Mendilaharsu et al., 2020). However, it is mainly in the tropical region of the Northeast where the reproductive areas are found (Marcovaldi & dei Marcovaldi, 1999; ICMBIO, 2016). Even though it is known that gillnet is the greatest threat to turtles, there are knowledge gaps in the region (Guebert, Barletta & Costa, 2013; Nogueira & Alves, 2016).

In 2021, Brazil signed an agreement with Global Fishing Watch to provide tracking data from industrial fishing vessels, but information is still lacking for artisanal vessels (Vosburgh, 2021). Moreover, it is widely known that the Brazilian government has not had reliable official data on fisheries for at least 10 years (Neto et al., 2021). Developing countries such as Brazil require urgent conservation actions even if biological data is scarce or insufficient to guide decision-making. Although a high value is placed on standardized, large-scale biological data collection in conservation, natural resource management has already been done even without scientific data by communities in self- and co-management systems worldwide for a long time (Ostrom, 2009; Castello, 2023).

One way to enable conservation to be carried out in data-poor areas is to conduct participatory research, (here defined as “systematic inquiry in direct collaboration with those affected by an issue being studied for the purpose of action or change” per Vaughn & Jacquez, 2020). Citizen science and local ecological knowledge are ways to contribute to scientific knowledge and field experience, addressing complex questions about social-ecological systems (*e.g.*, natural resource management, fishery management, species distribution, abundance and behavior) (McKinley et al., 2017; Bonney et al., 2021; Tengö et al., 2021). Citizen science consists of the public participation in scientific research and production, and has become popular with the availability of information technology infrastructures to process, organize and share data (McKinley et al., 2017; Fraisl et al., 2022). Local ecological knowledge is accumulated from the practical relationship that people have with the natural resources used. This relationship can be historical when transferred over generations or from observation and shared experience in the same generation (Narchi et al., 2014; Silvano et al., 2023). Folk taxonomy, for example, consists of naming and classifying organisms using a set of traditional values and empirical observations (Medeiros et al., 2022).

Involving local communities in monitoring allows knowledge to be produced for users and resource management at specific scales of time and space. It also allows that problems or fundamental issues in the locality to be addressed, which may not correspond to the issues identified by scientists or decision-makers (*e.g*., top-down approaches in conservation). Involving local stakeholders in monitoring has also been shown to improve management responses at the local scale, leading to faster and more cost-effective implementation of decisions, if compared to monitoring arrangements imposed by scientists (Castello, 2023). Participatory approaches make it possible to map specific small-scale fishing territories, for example, and provide important data to support the management, conservation, and ecology of marine species (Verutes et al., 2020; Silvano et al., 2023). Some benefits include the definition of marine protected areas (MPAs), the indication of distribution patterns of species and the creation of fishing rules (Silvano et al., 2023).

The use of geotechnologies applied to studies of marine megafauna, such as sea turtles, has increased (Verutes et al., 2020; Costanza et al., 2021), but it is still not common if compared to applications to forest ecosystems (Novo et al., 2005). The integration of remote sensing techniques and mathematical optimization with environmental data of significant biophysical characteristics, such as depth, distance from the coast, water transparency, surface temperature, and chlorophyll concentration in the ocean can be an important tool in the characterization of areas of occurrence of certain animal species (*e.g*., preferred habitats). Sea turtles have been shown to have a positive relationship with shallow waters with high chlorophyll concentration, as they are a proxy for productivity, foraging availability, and habitat selection (Kobayashi et al., 2008; McCarthy et al., 2010). Although turtles do not consume chlorophyll directly, many of their preferred prey may be more abundant in these areas, as indicated by studies with *Caretta caretta* in the North Pacific Ocean (Polovina et al., 2004; Kobayashi et al., 2008) and the North Atlantic (McCarthy et al., 2010), and *Lepidochelys olivacea* in the Bay of Bengal (Mishra et al., 2022). Studies that have not confirmed spatial correlation with chlorophyll concentration may be explained by dietary preference biases (Polovina et al., 2004; Roberts et al., 2021) or reproductive ecology (Behera et al., 2018). *Caretta caretta* preferentially feed on invertebrates which may cause them to avoid areas of high chlorophyll concentration and seagrasses (Roberts et al., 2021). Moreover, analyses of stomach contents of *L. olivacea* tracked in the North Pacific Ocean demonstrated that their prey was found in deeper areas than the shallow waters where chlorophyll concentration is higher (Polovina et al., 2004). In that case, time spent at the water surface may be due to post-nesting migrations or increasing body temperature by basking and not necessarily due to foraging (Behera et al., 2018).

Identifying areas of high use by sea turtles and mitigating the impacts of fishery bycatch are challenges, especially in the context of the wide range of small-scale fisheries in coastal communities. However, it must be considered for long-term conservation efforts (Hamann et al., 2010). These species become more sensitive to bycatch due to their population dynamics, specifically, they have later sexual maturity and low recruitment rates (Lutz, Musick & Wyneken, 2002), which can lead global population decline with frequent removal of adults (Lewison et al., 2004). Furthermore, with the increased intensity of fishing operations in high-abundance areas for turtles, mortality from accidental capture is expected to increase (Lewison et al., 2004; Putman, Hawkins & Gallaway, 2020). It makes urgent a better understanding of the implications of conservation efforts and regulations to achieve the target of bycatch reduction (Putman, Hawkins & Gallaway, 2020).

In this sense, there is a gap in the literature regarding turtle habitats in overlapping small-scale fishing areas in Northeastern Brazil. To our knowledge, this is the first study that aims to fill this gap. The overall aim of this study was to use ethnobiology and participatory approaches as simple and cost-effective methods to identify areas for sea turtle conservation where impacts from small-scale fisheries are most likely. To achieve this we aimed to (i) identify sea turtle species in interaction with small-scale fisheries, and (ii) predict their habitats and evaluate a degree of risk of interaction based on the spatialization and intensity of coastal fisheries. We used a participatory and complementary approach by integrating the local ecological knowledge of fishers and ecological modeling to provide information for decision-makers concerning endangered species conservation and marine spatial planning.

## Materials and Methods

### Study area

**Figure 1:**
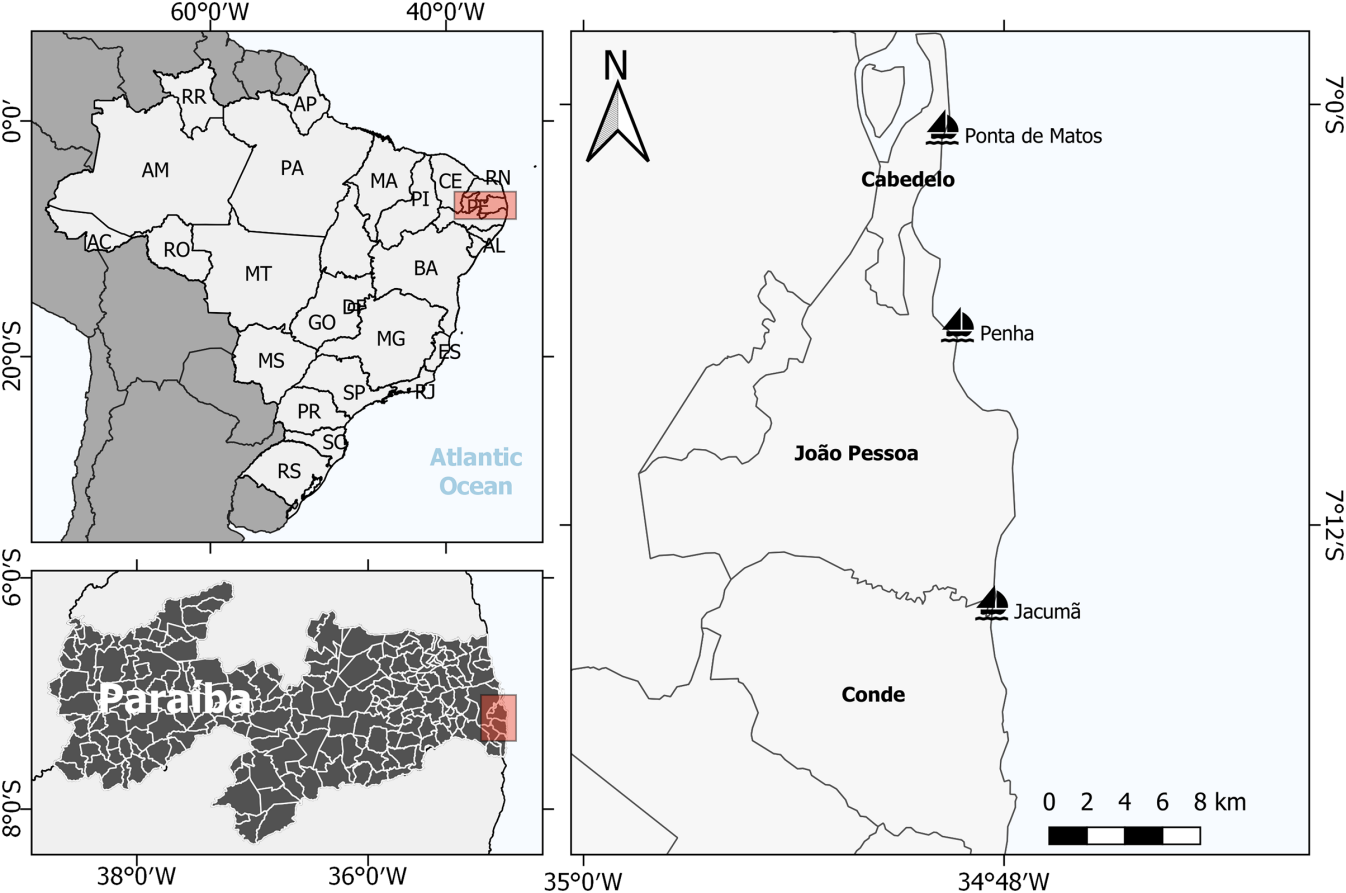
Studied areas in Paraiba, Northeast Brazil: Cabedelo, João Pessoa and Conde. Three fishing communities where volunteers were recruited: Ponta de Matos, Penha and Jacumã, respectively.

Located in northeastern Brazil, Paraíba state has 138 km of coastline (Fig. 1), where canoes and boats are traditional vessels of small-scale fishery. Gillnets are the main fishing gear used, and the studied communities are among the urban areas with the highest artisanal fishing productivity (Ponta de Matos, Penha and Jacumã) (IBAMA, 2005, FUNDAÇÃO PROZEE, 2006; Mariano & Rosa, 2010).

### Data collection and statistical analyses

Participant observation was done before beginning data collection at the three landing ports. Regular visits to the fishing villages to meet the community and their routine were ways to gain rapport with the fishers, building a relationship of trust between the potential survey participants and the researcher (Albuquerque et al., 2014; Awabdi et al., 2021).

We identified local experts using the snowball method, which is used with small-scale fishers in ethnobiology research and consists of a chain of referral among the survey participants (Albuquerque et al., 2014; Costanza et al., 2021; Martins et al., 2022). We asked fishers about the captains who had the most navigation experience (*i.e*., long years, fishing frequency) and knowledge about the main fishing grounds accessed in each community. We believed that the captains would provide more realistic information on the areas where sea turtle habitats and fishing activity overlapped.

We specifically selected nine captains from the landing ports at Ponta de Matos (*n* = 3), Penha (*n* = 3), and Jacumã (*n* = 3) to avoid duplicate information on the same vessel. However, all crew members have received training to help the captain to record sea turtle sightings (date, time) and ethnobiological data (*e.g.*, number of individuals, life stage, sex and folk name).

During navigation from the landing port to the fishing grounds, fishers on board the vessels recorded sightings of free-swimming turtles. Each vessel was tracked by the GPS logger tracker (I-gotU USB GPS Logger–GT600), which was programmed to receive geolocations at 5-min intervals (Vieira et al., 2017), working for 15 days and being replaced when discharged. Due to budget constraints, we only purchased nine GPS trackers. The daily records began in the early-morning hours and ended in the late afternoon. Captains were responsible for recording sea turtle sightings in a logbook, but all crew members could assist the captain to find the individuals.

We presented a board with photographs of the sea turtle species from Brazil to the fishers who recorded sightings at the species level. Photographs were at different angles, with submerged and surface images with the scientific name given on the back. It allowed us to assess the fisher’s ability to recognize the species (Awabdi et al., 2021) and the correspondence between the scientific and folk nomenclatures. Only three fishers recorded sightings as “turtle”, which did not impact the correspondence test.

Comparisons between three communities were made by a one-way ANOVA followed by Tukey’s *post hoc* multiple comparison test. Fully-structured interviews were conducted to assess the socio-economic attributes of fishers (*e.g.*, target species, fishing gear, fishing frequency, source of income, vessel type). Fishers were informed verbally about the Free Prior and Informed Consent to authorize the use of information provided for the research. Participant observation, interviews and field sampling were carried out from June to November 2018.

### Identifying sighting points and fishing areas

**Figure 2:**
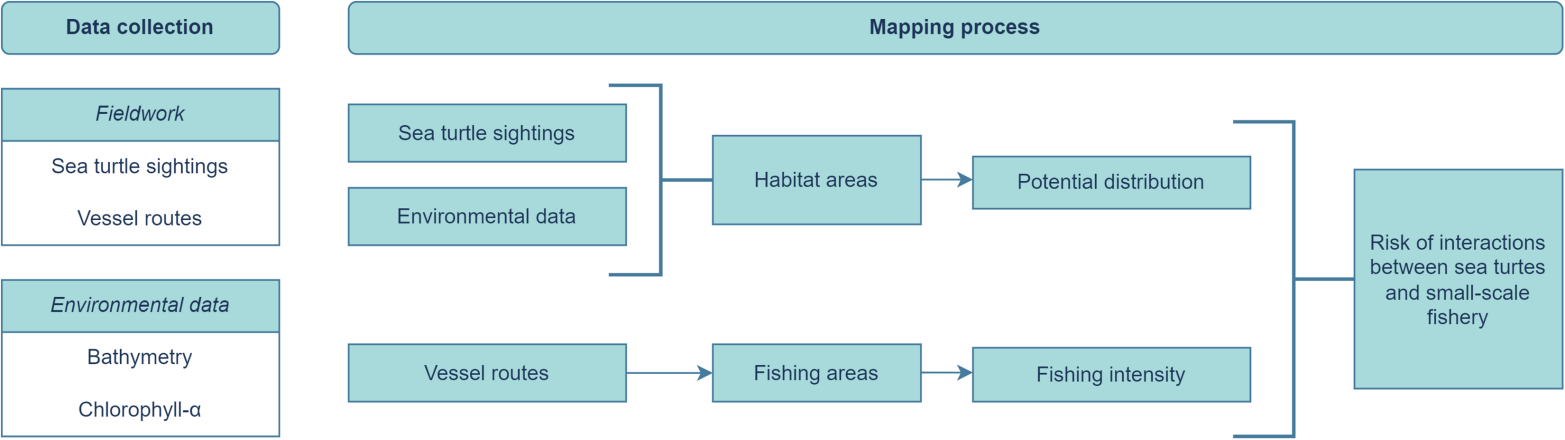
Methodological approach. Data collection and mapping process for plotting the risk of interactions between sea turtles and small-scale fishery.

The data from the daily route of the vessel was extracted from the GPS, with a Python script (see Supplemental Material) that allowed for the identification of sighting points and fishing areas. Sighting points were defined based on the association of date and time information collected in the logbook by the fishers and the GPS position (Fig. 2). However, due to the small sample size, it was not possible to produce species-specific analysis as such we maintained all sighting information on the analyses. Fishing areas were defined as the minimum convex polygon containing all GPS information for all vessel routes, separated between the communities (Fig. 2).

### Environmental data and area of habitat prediction

The environmental data used were surface reflectance as an indirect measure of surface chlorophyll-α for coastal waters (Chan, Fan & Yao, 2022) and the bathymetry data from the Bathymetry Project (Ladeira Neto & Roza, 2013) was used to help define a physical barrier to the distribution of turtle species (Kobayashi et al., 2008; McCarthy et al., 2010). Surface reflectance is the amount of light reflected by the surface of the earth, for this article we choose band eight of MODIS/Terra MODOCGA (NASA, 2022) which provides daily measurements with a spatial resolution of 1 km (Vermote & Wolfe, 2015), and can be used in studies of concentration of chlorophyll-α in coastal waters (Longhurst & Glen Harrison, 1989; Groom et al., 2019).

For each day of the sightings, we extracted the daily measurements from MODOCGA for each of the sighting points and selected all pixels for that day with the same value. From the integration of all data, a layer was obtained for each day of observation and the “Habitat Area” (*i.e*., areas with similar environmental conditions to the sighting sites) was delimited. We also calculated “Potential Distribution”, by following the steps below:

1. A 1 km² grid was created (equivalent to the spatial resolution of the MODIS/Terra MODOCGA product);

2. The areas of each daily layer were identified in the pixels inside the mesh and transformed into centroids with a value of 1;

3. With the “spatial join” process, the sum total of values for spatially correlated pixels within the 1 km² grid with the centroids of the daily layers was calculated.

This value indicated an index of the potential distribution of sea turtles per area, representing the sum of the number of times that a location appeared in each of the layers, for each day of sighting, ranging from 0 to 9. Allied with the analysis of habitat areas, a map was prepared with an index of the potential distribution of sea turtles within these areas.

### Mapping risk of fishery interaction with sea turtles

We created degrees of fishing intensity defined by the concentration of vessel routes in each community. The areas with the greatest intersections of routes in the 1 km² window were considered as having a higher probability of impact on sea turtles. We have a data variation between 0 and 71 fishing trips at the sites. Since we did not have a normal distribution for the fishing intensity with the raw data and neither by adding the constant one to the log-transformed data, we used the bootstrap based on 1,000 replications to calculate the confidence intervals at the 95% level. Raw data: 0.3908, 0.5593; log-transformed data: 0.2034, 0.2360. Analyses were performed in R software, version 4.2.3 (R Core Team, 2023), using the “boot” package (Canty & Ripley, 2022).

“Risk of interaction” is defined as the risk of entanglement or vessel collisions, calculated by multiplying the “Potential Distribution” with “Fishing Intensity” made in the same spatial grid with 1 km² as the “Habitat Area” (Verutes et al., 2020; Costanza et al., 2021). To ensure that both “Potential Distribution” and “Fishing Intensity” were on a similar scale and given the same weight, they were both converted to a base 10 logarithmic scale. This allowed them to be accurately compared and combined to create the ‘Risk of Interaction’ score, using the Inverse Distance Weighting method (IDW; Bartier & Keller, 1996) we extrapolated the results for the entirety of the “Habitat Area” based on the distance to the calculated value.

## Results

### Ethnobiological data

We recorded a total of 218 sightings, of which 51.37% (*n* = 112) were not identified at the species level by the fishers, being noted only as “turtle”. Among records with a species-level identification (*n* = 106), most of our records were from “tartaruga-verde” (*C. mydas*, *n* = 88), which can be explained both by their behaviour of using the coastal habitat and by the ease of morphological identification (shape and colour of the carapace and head). “Tartaruga-cabeçuda” (*Caretta caretta, n* = 11) and “tartaruga-de-pente” (*Eretmochelys imbricata, n* = 7) were less recorded, although this identification was difficult even for the researchers since the sightings were about 5 m away from the boat. Adults (47.20%) were sighted more than juveniles (13.30%), and identification of sex occurred only for males (*n* = 2).

There was a significant difference in correspondence of folk and scientific taxonomy between fishing landing sites (*p* < 0.05), where classification match in Ponta de Matos and Penha (75%) was higher than in Jacumã (45%).

Most of our records (61%) were from Ponta de Matos as a result of the greater engagement of these fishers. All communities had similarity in the gears used and target species in the fisheries. All the fishers (*n* = 9) reported using gillnets in the catches of important reef fishery resources (Medeiros et al., 2018): “robalo” (*Centropomus undecimalis*), “chicharro” *(Selar crumenophthalmus*), “cioba” (*Lutjanus analis*) and “cavala” (*Scomberomorus cavalla*). Only one fisher also reported using a line and hook.

However, there were differences between the dependency on fishing activity for income between communities. In Jacumã, all fishers have fishing as their only source of income, which did not occur in Ponta de Matos (33.33%) and Penha (66.67%). Although all were characterized as small-scale fishers, a motorboat was common only in the Jacumã fishing fleet. In the others, rafts were usually present (Fig. S1). Table 1 shows the fishers’ effort for recording sea turtle sightings from each landing port.

**Table 1:** Fishers’ efforts to record turtle sightings in coastal communities of Paraiba, Northeastern Brazil.

Fisher ID	Sightings (*n*)	Effort (days)	Landing port
1	65	16	Ponta de Matos
2	7	2	Ponta de Matos
3	61	6	Ponta de Matos
4	5	1	Penha
5	22	7	Penha
6	7	2	Penha
7	30	10	Jacumã
8	19	3	Jacumã
9	2	2	Jacumã

### Sea turtle habitat and fishing areas

We have identified sea turtle habitats in shallow reefs of up to 50 m on the coast of Paraíba, overlapping with areas used by small-scale fishers. A total of 192 sea turtle sightings were recorded, corresponding to 42 daily images from MODIS/Terra MODOCGA. Some daily data were not available for the region, so only 28 daily images were analyzed. Sea turtle habitats were delineated by a bounding box (delimiting polygon surrounding the sighting points).

**Figure 3:**
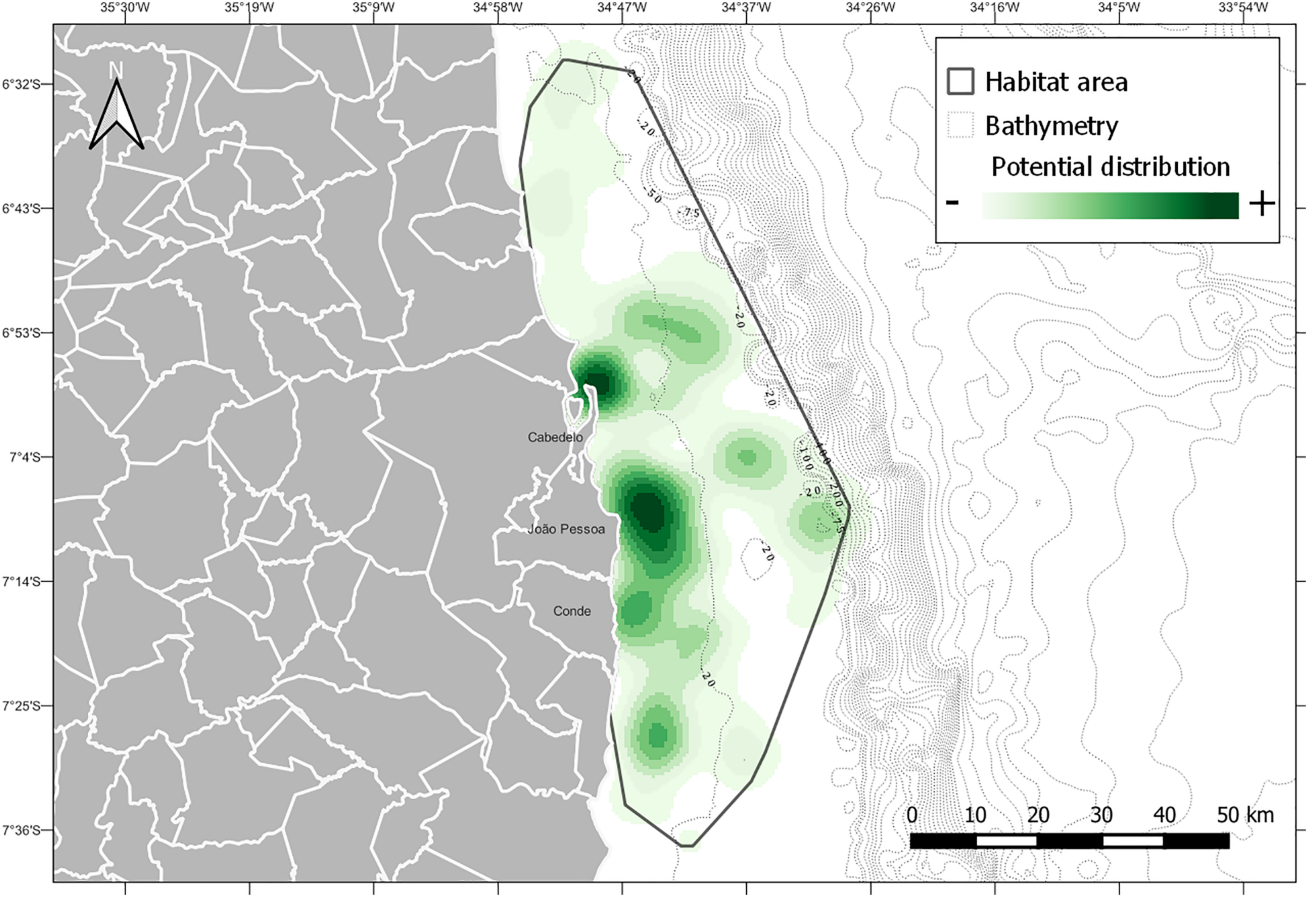
Habitat areas of sea turtles along the Paraiba coastline. Modeling was obtained using ethnobiological and environmental (chlorophyll and bathymetry) datasets from NASA (2022) and CPRM at https://www.cprm.gov.br/publique/Geologia/Geologia-Marinha/Projeto-Batimetria-3224.html. Bathymetry is represented by black dotted line. Potential distribution is represented in degrees from lowest (white) to highest (green) occurrence.

Using the 28 remote sensing images, 3,459.96 km² of habitat areas for sea turtles on the coast of Paraíba were identified. Based on the occurrence sites, the habitat areas corresponded to locations with this same pattern of reflectance data (*i.e*., data regarding the concentration of chlorophyll in the ocean). Chlorophyll concentration was directly related to the gradient of green, brown and calcareous algae and macroalgae, and phytoplankton community. The prediction of habitats for sea turtles based on the concentration of chlorophyll in the ocean related to the availability of resources and feeding zones for the species. Allied with the analysis of habitat areas, a map was prepared with an index of the potential distribution of sea turtles within these areas (Fig 3).

**Figure 4:**
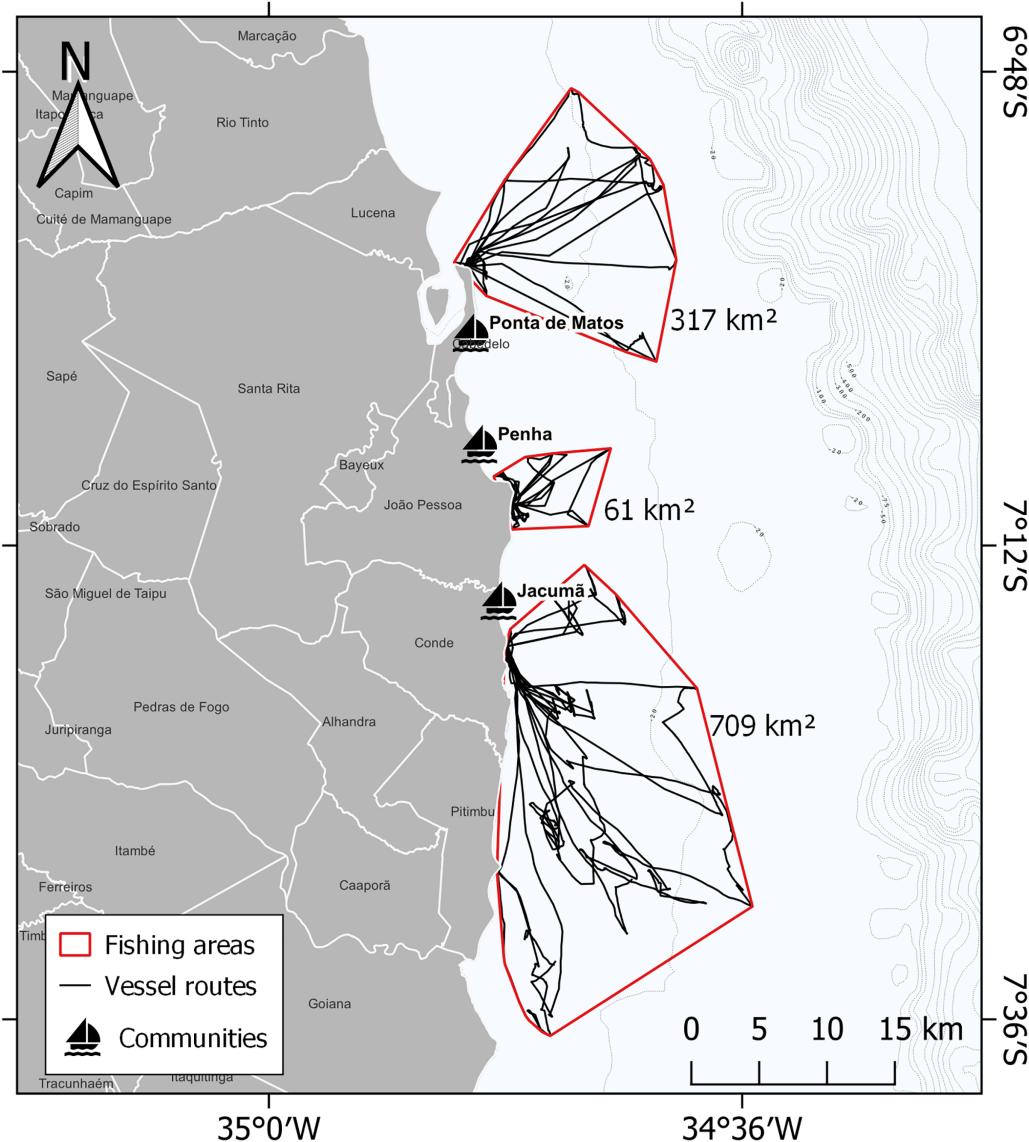
Fishing areas used by three communities studied: Ponta de Matos in Cabedelo, Penha in João Pessoa and Jacumã in Conde. This model output the fishing areas in red polygons and vessel routes are represented by black lines.

**Figure 5:**
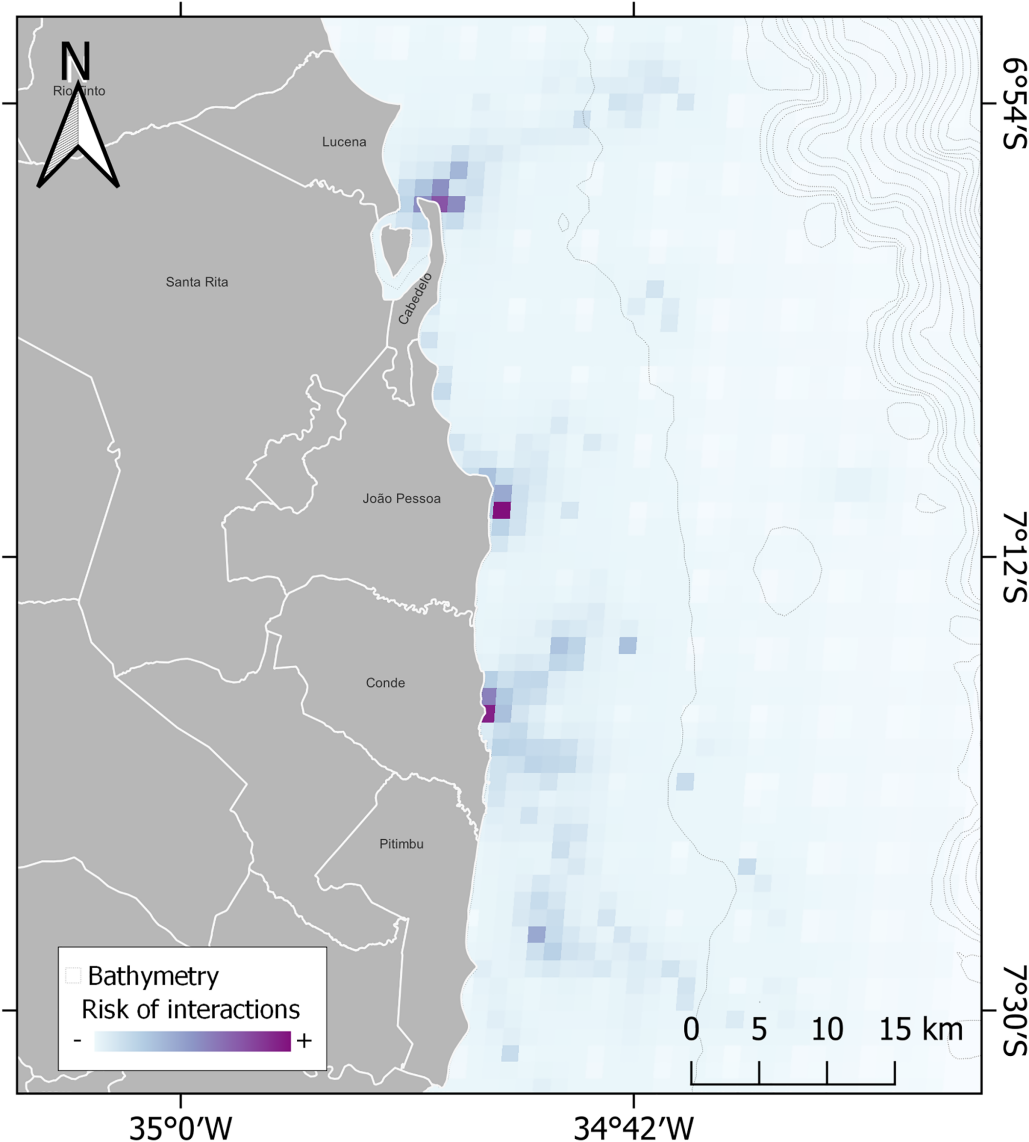
Modelling of risk of interactions between sea turtles and small-scale fishery. Interpolation was obtained using the habitat and fishing areas dataset whose environmental data are from NASA (2022) and CPRM at https://www.cprm.gov.br/publique/Geologia/Geologia-Marinha/Projeto-Batimetria-3224.html. Bathymetry is represented by black dotted solid lines. Potential distribution is represented in degrees from minor risk (white) to major (purple).

Summing the areas in use by the three communities, there was a total of 1,087 km² on the coast of Paraiba up to the continental shelf, with Jacumã having the greatest fishing use area (Fig. 4). In Fig. 5, we have identified a high risk of interaction near landing ports and coastal habitats for sea turtles species.

## Discussion

Our study addresses, for the first time, information about specific areas that may have high fishing impacts on sea turtles in Northeast Brazil. It may help to improve regulations in small-scale fisheries to mitigate threats. For example, the full protection from extractive activity in fishing grounds (*e.g*., no take zones) may generate negative effects in the livelihood of fishers and reinforce overexploitation. The co-management of marine resources (*i.e*., natural resource management shared between social and private actors; Carlsson & Berkes, 2005) can help to reach ecological and social goals in social-ecological systems, but the level of dependence on fishing for income is a key factor for the successful outcomes (Cinner et al., 2012).

We also address important issues: (i) fishers’ knowledge about sea turtle occurrence helps to predict their preferred habitats and highlights risks of interaction in overlapping areas (*e.g.*, foraging grounds and fishing areas), and (ii) it is a low-cost method to identify fishing areas in locations with poor or absent data on small-scale fisheries.

### Mapping critical areas for sea turtles

Aggregations of *C. mydas* found mainly up to 20 m depth were expected and can be explained by the results also found by Santos et al. (2015) regarding its foraging behaviour on the Brazilian coast. Our habitat area results are also consistent with a previous study on the diving behaviour of loggerhead and green turtles that show that they spend most of their time at depths of up to 100 m (Fiedler et al., 2012). In tropical reefs, green turtles have a benthic herbivore foraging pattern (Santos et al., 2015), which is consistent with the feeding habits on the Paraiba coast. Even in urban and temperate zones of the Pacific Ocean, seagrass pastures prove to be an important resource. Knowledge about the distribution of prey species indirectly provides information on foraging habitats preferred by *C. mydas* (Lemons et al., 2011), which could increase the available information if complemented by data collection with the use of geotechnologies. Coastal fisheries overlap mainly green turtles habitats due to their coastal patterns. Besides the risk of bycatch, gillnets also lead to the animals’ mortality indirectly, by facilitating the entry of infectious agents through the injuries caused by the gear (Domiciano, Domit & Bracarense, 2017).

Similar to other sea turtle species, *E. imbricata* has philopatric behavior and has regular spawning areas in Paraiba (Simões et al., 2021). They also use these tropical waters as feeding grounds and play an important role in reef structure and dynamics (León & Bjorndal, 2002). *Caretta caretta* are considered abundant on the coast of Brazil, and they have reproductive and ecological similarities to *E. imbricata*, such as the number of hatchlings, nests and similar spatiotemporal reproductive periods (Relvas et al., 2022). Some studies applied to remote sensing and mathematical optimization have allowed to identify species’ area of habitat (Brooks et al., 2019), monitor wildlife (Behera et al., 2018), and designate MPAs (Kachelriess et al., 2014). A global review of satellite telemetry studies on green turtles (Scott et al., 2012) identified coverage of marine protected areas in aggregation areas of this species. As the telemetry tool is expensive, our work presents a low-cost and feasible alternative to map habitat use and aggregations, not only for sea turtles but it may also be replicable for other megafauna species, which are easily sighted by local communities.

### Conservation efforts benefit nature and small-scale fishers

Our method also allows identification of important areas for small-scale fisheries and can guide decision-making for marine spatial planning. Generally, marine protected areas are established for the conservation of a particular species or ecosystem without taking into account the socio-ecological aspects of the system (Cinner et al., 2012). Such a top-down approach usually involves conflicts with users that are intrinsically dependent on these environments, which become closed with prohibition or restriction of access to their natural resources (Cinner et al., 2012; Karnad, 2022). In this sense, by participatory mapping of threatened species and marine territories used by fishers, management agreements that depend on the regulatory compliance of MPA users, as well as their perception of conservation objectives, can be facilitated (Karnad, 2022). The approach of justice and socio-environmental management present in political ecology can avoid situations of worsening persecution of marginalized populations without neglecting the conservationist purpose (Aswani et al., 2018).

When spatial or temporal restrictions on fishing are not adequate for MPAs, the bycatch reduction technologies may contribute to controlling the negative impacts of fisheries. Using green light (Wang, Fisler & Swimmer, 2010) or ultraviolet light (Wang et al., 2013) on gillnets can reduce incidental sea turtle catches by up to 63.9% (Ortiz et al., 2016), without affecting the catch of the target species. In addition, this bycatch reduction technology can be multi-taxa, avoiding the capture of seabirds as well (Mangel et al., 2018). Identifying where impacts of small-scale fishery are most likely, such as through participatory mapping, may help to define specific areas for combining appropriate strategies to mitigate threats. Moreover, the interaction of turtles with fisheries also results in financial loss for fishers (*e.g*., gear loss or destruction), which may ease the adoption of bycatch reduction technologies and economic incentives (*e.g*., eco-labeling and certification) encouraged by public policies (Gandini & Frere, 2012; Lent & Squires, 2017).

As is traditional in small-scale fisheries in Brazil (Guebert, Barletta & Costa, 2013), the three fishing communities in Paraiba use mainly gillnets. This gear is known for its high risk of causing accidental captures, along with trawl and pelagic longline (Lewison et al., 2004; Panagopoulou et al., 2017; Awabdi et al., 2021). In a study concerning the impact caused by Peruvian small-scale fisheries on turtle species, the mortality rates presented for gillnet catches were very high (41%). This magnitude could still be underestimated in a region with weak species protection measures and monitoring of fishing activity, which already has a diffuse effort (Alfaro-Shigueto et al., 2011).

Among the monitored areas, the fishery in Jacumã has the greatest potential of fishing interaction with sea turtles, based on the use of 709 km^2^, which is about eleven times larger than Penha, for example. This result highlights the importance of knowing the socioeconomic aspects of fishers, and the need for fishery characterization to obtain a greater dimension of the pressure on the stocks of target and accessory species. Mapping fishing routes with small-scale fishers is a simple and cost-effective way to increase knowledge about the use of fishing areas and predict potential impacts for species in overlapping areas. Furthermore, it provides relevant information for understanding the socioecological dynamics, which can be fundamental to meet social and ecological goals in co-management systems (Cinner et al., 2012). Similarly, for other threatened marine species (Lopes et al., 2019; Costanza et al., 2021), participatory GIS has proven to be a viable alternative for gathering information in developing countries that lack reliable information for decision-making on the management and conservation of sea turtles.

The weakness in species recognition in Jacumã may be explained by the (i) isolation of the community compared to the others, (ii) wide fishing zones that reduce the number of encounters with marine vertebrates, and (iii) limitation of the visual field on board due to equipped with a cabin, such as motorboats (Fig. S1).

### Limitations and future directions

Limitations of this work involve the difference in sampling effort among fishers and the low number of fishers engaged in data collection, even though it was enough to provide relevant information on species occurrence, local ecological knowledge and threats. For future studies, a larger sample considering seasonal dynamics of fisheries and more localities is recommended, as well as detailing deeper layers of ethnobiological data. This study comprised the winter months, but fishing occurs all year round, with different target-species varying along seasons. In an evaluation of the interactions of juvenile green turtles with gillnets in Southern Brazil, it was found that the highest capture rates occurred at the end of the rainy season, although the level of mortality was higher at the beginning of the dry season. In this area, the dry season corresponds to the coldest period of the year, which could explain the animals’ physical restrictions at low temperatures (López-Barrera, Longo & Monteiro-filho, 2012). In contrast, the period with the lowest temperatures in our study area occurs during the rainy season. This highlights the need for a better understanding of the dynamics of bycatch and mortality due to small-scale fishery throughout the year in this region. We recommend gathering information on bycatches and strandings from fishing communities with a long-standing relationship of trust with the research team.

Characterization of fisheries from a socioeconomic perspective can be important to understand the role of fishing in the subsistence of families, which will influence effort (*e.g.*, more or fewer days spent at sea), gear, target species of capture and pressures on accessory species. In coastal fisheries along the southeastern coast of the USA, higher bycatch rates of juvenile sea turtles were found in recreational fisheries, which indicates the importance of considering other fishing categories in future studies (Putman et al., 2023). Although expensive, the satellite telemetry studies would complement the information acquired by participatory monitoring, broadening the knowledge of how individuals use areas with fishing activity. It is also recommended that temperature be considered among the environmental data for satellite remote sensing since it proved to be relevant for modeling the occurrence and distribution of species (Lopes et al., 2019; Costanza et al., 2021). In a global review of *Chelonia mydas* diet, the temperature of the sea surface has also shown to influence its dietary preference. In warm areas, with temperatures above 25 °C, the diet was herbivorous, while sites with colder currents tended to influence omnivory (Esteban et al., 2020).

## Conclusions

This work contributes to ongoing discussions of bycatch mitigation for threatened and migratory species in Northeast Brazil and highlights the importance of considering particularities of overlapped small-scale fishing areas in marine spatial planning. Our study showed for the first time that fishers’ knowledge combined with participatory mapping can provide information about sea turtle habitats and fishing areas in a data-poor context. Our results reinforce the notion that ethnobiological data provides relevant information on species occurrence and support their distribution modeling. Even with little data from fishers, it was possible to find priority areas to help guide immediate actions related to the mitigation of fishery impacts on sea turtles. Furthermore, our approach is feasible to be used in countries with a lack information to help as a source of knowledge that can lead to changes in management of their endangered species. Considering that knowledge about fishing zones is required to ensure that socioeconomic goals are also achieved in marine spatial planning, conservation efforts must be directed toward the strengthening of co-management structures, such as avoiding overexploitation or inequity and improving fishers’ compliance to reduce threats to sea turtles.

## Supplemental Information

10.7717/peerj.17109/supp-1 Supplemental Information 1 Category of vessels used the fishing communities Ponta de Matos, Penha e Jacumã. (A) Raft. (B) Canoe. (C) Motorboat. (D) raft, canoe and boat in a fishing port, from left to right.

10.7717/peerj.17109/supp-2 Supplemental Information 2 Area of habitat of sea turtle species (*Caretta caretta*, *Chelonia mydas* and *Eretmochelys imbricata*) along the Paraiba coastline. Modelling was obtained using the ethnobiological and environmental (chlorophyll and bathymetry) dataset. Bathymetry is represented by grey solid lines. Projected occurences are represented by circles and sightings are represented by black points.

10.7717/peerj.17109/supp-3 Supplemental Information 3 Mapping data including vessel routes of fishing communities in Paraiba, Brazil.

10.7717/peerj.17109/supp-4 Supplemental Information 4 R script (boot package) used in the analysis of fishing intensity data.

10.7717/peerj.17109/supp-5 Supplemental Information 5 GPX files from vessel routes of nine fishers recorded between July and November 2018 in study area.

10.7717/peerj.17109/supp-6 Supplemental Information 6 Python script to identify sighting points from route files (GPX - GPS Exchange Format), migrated from tracker devices (I-gotU USB GPS Logger – GT600), loaded and run in QGIS software interface environment.

## Additional Information and Declarations

### Data Availability

The following information was supplied regarding data availability: The SIG data is available at FigShare: Bezerra Barbosa de Oliveira, Yedda Christina (2023). Participatory GIS data. figshare. Dataset. https://doi.org/10.6084/m9.figshare.21957002.v1.
