# Supplementary figures and images for: Critical areas for sea turtles in Northeast Brazil: a participatory approach for a data-poor context
Source: PeerJ. 2024 Mar 25;12:e17109. doi: 10.7717/peerj.17109 (PMC10977088; doi:10.7717/peerj.17109)

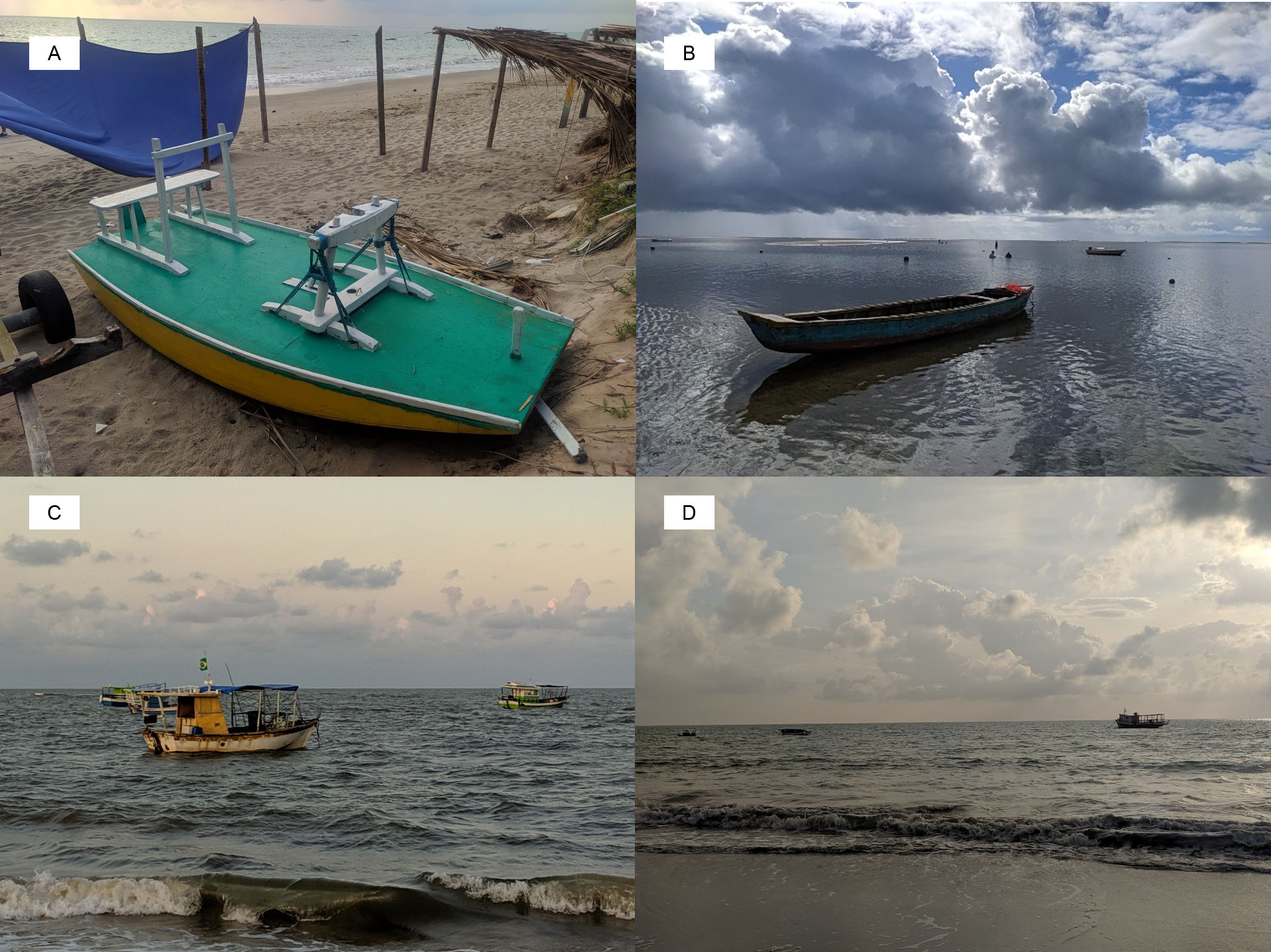

Supplement: Supplemental Information 1 — (A) Raft. (B) Canoe. (C) Motorboat. (D) raft, canoe and boat in a fishing port, from left to right. [file peerj-12-17109-s001.png]

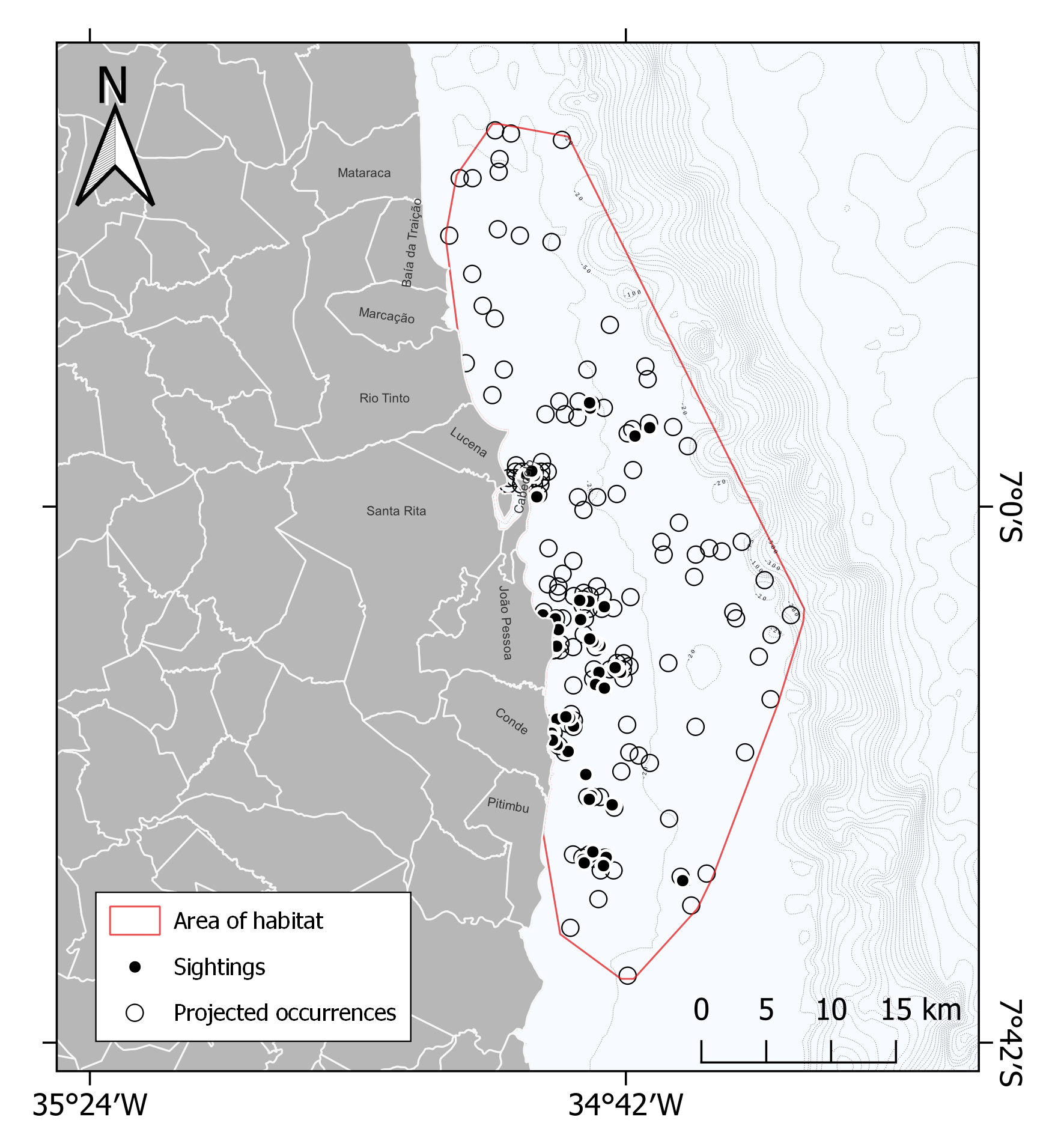

Supplement: Supplemental Information 2 — Modelling was obtained using the ethnobiological and environmental (chlorophyll and bathymetry) dataset. Bathymetry is represented by grey solid lines. Projected occurences are represented by circles and sightings are represented by black points. [file peerj-12-17109-s002.png]
